# Patients with obstructive sleep apnea can favor the predisposing factors of periodontitis by the presence of *P. melaninogenica* and *C. albicans*, increasing the severity of the periodontal disease

**DOI:** 10.3389/fcimb.2022.934298

**Published:** 2022-09-15

**Authors:** Mayra A. Téllez-Corral, Eddy Herrera-Daza, Hayde K. Cuervo-Jimenez, Natalia Arango-Jimenez, Darena Z. Morales-Vera, Juliana Velosa-Porras, Catalina Latorre-Uriza, Francina M. Escobar-Arregoces, Patricia Hidalgo-Martinez, Maria E. Cortés, Nelly S. Roa-Molina, Liliana Otero, Claudia M. Parra-Giraldo

**Affiliations:** ^1^ Centro de Investigaciones Odontológicas, Facultad de Odontología, Pontificia Universidad Javeriana, Bogotá D.C., Colombia; ^2^ Unidad de Investigación en Proteómica y Micosis Humanas, Facultad de Ciencias, Pontificia Universidad Javeriana, Bogotá D.C., Colombia; ^3^ Facultade de Odontología, Programa de Pós-graduação em Inovação Tecnológica, Universidade Federal de Minas Gerais, Belo Horizonte, Minas Gerais, Brazil; ^4^ Departamento de Matemáticas, Facultad de Ciencias, Pontificia Universidad Javeriana, Bogotá D.C., Colombia; ^5^ Periodoncia, Facultad de Odontología, Pontificia Universidad Javeriana, Bogotá D.C., Colombia; ^6^ Clínica del Sueño, Hospital Universitario San Ignacio y Facultad de Medicina, Pontificia Universidad Javeriana, Bogotá D.C., Colombia

**Keywords:** cultivable oral microbiota, periodontitis, obstructive sleep apnea, MALDI-TOF-MS, *Candida albicans*

## Abstract

**Objective:**

The aim of this study was to analyze the cultivable oral microbiota of patients with obstructive sleep apnea (OSA) and its association with the periodontal condition.

**Methods:**

The epidemiology profile of patients and their clinical oral characteristics were determined. The microbiota was collected from saliva, subgingival plaque, and gingival sulcus of 93 patients classified into four groups according to the periodontal and clinical diagnosis: Group 1 (*n* = 25), healthy patients; Group 2 (*n* = 17), patients with periodontitis and without OSA; Group 3 (*n* = 19), patients with OSA and without periodontitis; and Group 4 (*n* = 32), patients with periodontitis and OSA. Microbiological samples were cultured, classified, characterized macroscopically and microscopically, and identified by MALDI-TOF-MS. The distribution of complexes and categories of microorganisms and correlations were established for inter- and intra-group of patients and statistically evaluated using the Spearman *r* test (*p*-value <0.5) and a multidimensional grouping analysis.

**Result:**

There was no evidence between the severity of OSA and periodontitis (*p* = 0.2813). However, there is a relationship between the stage of periodontitis and OSA (*p* = 0.0157), with stage III periodontitis being the one with the highest presence in patients with severe OSA (prevalence of 75%; *p* = 0.0157), with more cases in men. The greatest distribution of the complexes and categories was found in oral samples of patients with periodontitis and OSA (Group 4 P-OSA); even *Candida* spp. were more prevalent in these patients. Periodontitis and OSA are associated with comorbidities and oral conditions, and the microorganisms of the orange and red complexes participate in this association. The formation of the dysbiotic biofilm was mainly related to the presence of these complexes in association with *Candida* spp.

**Conclusion:**

Periodontopathogenic bacteria of the orange complex, such as *Prevotella melaninogenica*, and the yeast *Candida albicans*, altered the cultivable oral microbiota of patients with periodontitis and OSA in terms of diversity, possibly increasing the severity of periodontal disease. The link between yeasts and periodontopathogenic bacteria could help explain why people with severe OSA have such a high risk of stage III periodontitis. Antimicrobial approaches for treating periodontitis in individuals with OSA could be investigated *in vitro* using polymicrobial biofilms, according to our findings.

## Introduction

Periodontitis is a chronic infectious disease that affects approximately 50% of adults in the world (Genco and Sanz, 2020). This infection is caused by pathogenic microbial communities known as dysbiotic microbiota. The dysbiotic microbiota produce an inflammatory response of the periodontal tissue resulting in the formation of periodontal pockets, progressive loss of periodontal attachment with the destruction of soft and hard supporting periodontal tissues, and loss of teeth (Hajishengallis and Korostoff, 2017).

This dysbiotic microbiota was described by Socransky (Socransky et al., 1998) who identified genera of microorganisms with certain pathogenicity factors that facilitate the colonization of periodontal tissue. In this way, he proposed six microbial complexes present in subgingival plaque recognized by colors: the yellow complex (*Streptococcus* spp.), the blue complex (*Actinomyces* spp.), the purple complex (*Veillonella* spp.), the green complex (*Eikenella* spp., *Capnocytophaga* spp., and *Campylobacter* spp.), the orange complex (*Eubacterium* sp., *Fusobacterium* spp., *Parviomonas* sp., *Prevotella* spp., *Slackia* sp., and *S. constellatus*), and the red complex (*Porphyromonas* sp., *Tanarella* sp., and *Treponema* sp.). The last two complexes are recognized for grouping the bacteria considered periodontal–pathogenic. Therefore, the periodontal–pathogenic dysbiotic microbiota, which is mostly bacterial, is strongly related to periodontitis as a determining factor in the inflammatory reaction of the periodontal tissues that results in systemic inflammatory events. (Lamont et al., 2018), triggering an exacerbated chronic inflammatory response, which is associated with systemic diseases, such as cardiovascular diseases (CVDs) (Bui et al., 2019), diabetes mellitus (Escobar et al., 2014), and obstructive sleep apnea (OSA) (Gamsiz-Isik et al., 2017).

OSA is a sleep-disordered breathing that occurs when the soft tissues around the upper airway collapse, partially or completely obstructing airflow despite the increased ventilatory effort. It is probably the most common sleep respiratory disorder in adults. OSA is more prevalent in men, and it becomes similar in both sexes after menopause (Ruiz et al., 2016; Hidalgo-Martínez and Lobelo, 2017). In recent years, OSA has been related to periodontitis. Previous studies have shown that people with OSA have a higher risk of severe periodontitis than people without sleep apnea and that periodontitis is more frequent in young adults with mild OSA (Gunaratnam et al., 2009; Seo et al., 2013; Sanders et al., 2015; Cuervo et al., 2016).

Different mechanisms involving genetic, immunological, and microbiological factors (Al-Jewair et al., 2015; Cade et al., 2016; Nizam et al., 2016; Zhang et al., 2021) have been suggested for the cause–effect relationship between OSA and periodontitis. The genetic predisposition, the inflammatory response shared in both diseases (Nizam et al., 2016), and the oral dryness (Seo et al., 2013) have been described among the hypotheses proposed to explain the increase in periodontitis in patients with OSA. Oral dryness decreases the ability of the immune system to respond to infections, alters bone remodeling stimulated by hypoxia, and increases CO_2_ levels (Nizam et al., 2016), enabling an environment that allows the colonization of a different polymicrobial and dysbiotic microbiota, in such a way that there may be a synergistic effect among these factors. Consequently, environmental changes in the oral cavity due to the relationship between periodontitis and OSA would allow associations of bacteria and yeasts (Vieira Colombo et al., 2016; Janus et al., 2017), which can form polymicrobial biofilms in the gingival sulcus. An *in vitro* study showed that *Porphyromona gingivalis*’ InlJ protein and *Candida albicans*’ hyphal protein Als3 facilitate the adhesion between the two microorganisms and cause significant changes in gene expression by *P. gingivalis*, thereby increasing the pathogenic potential of this bacteria (Sztukowska et al., 2018). In addition, a study revealed that *C. albicans* biofilms encourage the growth of anaerobic bacteria by removing the oxygen that is present and creating a hypoxic microenvironment (Fox et al., 2014).

Therefore, the presence of *Candida* spp. could play an important role in the progression of periodontitis due to its ability to colonize gingival tissue in greater proportion (Tamai et al., 2011; Canabarro et al., 2013; Arumugam, 2015). However, some authors still consider that there is little evidence for the association between periodontitis and OSA, the pathophysiological mechanisms, and the cause–effect relationship between both pathologies (Lembo et al., 2021). Then, it is necessary to start with a periodontal clinic analysis of the microbiological aspects present in patients with these pathologies and their relationship with the periodontal condition and the systemic factors that the patients are exposed to.

Proteomic tools such as Matrix-Assisted Laser Desorption/Ionization Time-of-Flight Mass Spectrometry (MALDI-TOF MS) allow the identification of microorganisms from libraries that store the mass spectrum of their cytoplasmic proteins, especially chaperones and ribosomal proteins. This technology allows the quick identification of up to 200 microorganisms as bacteria and yeasts, both aerobic and anaerobic (Barberis et al., 2014; Antezack et al., 2020). In this study we used MALDI-TOF MS to determine the microbial composition of cultivable oral microbiota (yeast and bacteria) in patients with OSA and its association with the periodontal condition.

## Materials and methods

### Patients/study population

A convenience sample of 93 eligible participants that fulfilled the inclusion criteria (56 women and 37 men aged 30–72 years) were enrolled from the Sleep Clinic of the *Hospital Universitario San Ignacio* and the Sleep Clinic of the Faculty of Dentistry at the *Pontificia Universidad Javeriana-PUJ*, Bogotá, D.C., Colombia with a polysomnographic diagnosis for OSA, and were included in the present study between April 2019 and March 2021. This study was carried out following the Declaration of Helsinki of 1975 revised in 2000 and approved by the Research and Ethics Committee of the Faculty of Dentistry at the *Pontificia Universidad Javeriana-PUJ* (CIEFOUJ 016B). Informed consent was obtained from all patients after the explanation of conditions and before their clinical examination.

The inclusion criteria were as follows: (1) adults over 30 years old; (2) having at least six teeth in their mouth; and (3) having a polysomnographic study done no more than 6 months before. The exclusion criteria were as follows: (1) smoker; (2) diabetic; (3) having taken antibiotics in the last 3 months; (4) no previous periodontal treatment in the last 3 months; (5) treated with continuous positive airway pressure (CPAP) or bilevel positive airway pressure (BPAP); and (6) having received a pharmacological or surgical treatment for OSA.

All patients were diagnosed by a sleep medicine pulmonologist. The apnea–hypopnea index (AHI) described in the polysomnogram was used to determine the presence and severity of OSA. AHI was calculated as the total number of apneas and hypopneas per hour of sleep. The occurrence of OSA was determined with an AHI score >5; mild OSA with an AHI of 5–15; moderate OSA with an AHI of 16–30; and severe OSA with an AHI >30 (Sateia, 2014). To determine the presence of periodontitis, all patients were examined at the clinic of the Faculty of Dentistry of the PUJ. Panoramic x-rays of each patient were taken and then the specialists in periodontics followed the same protocol to make the periodontal diagnosis of periodontitis stages I, II, III, and IV according to the 2017 World Workshop on the Classification of Periodontal and Peri-implant Diseases and Conditions (Caton et al., 2018). Periodontal probing was performed by two calibrated periodontists who used a North Carolina (Hu-Friedy^®^) probe to determine the insertion level, the gingival margin, and the pocket depth. Data from six points around the tooth were collected and bleeding with probing was determined. The presence of biofilm was determined with the O’Leary index (O’Leary et al., 1972). Patients were considered periodontally healthy if they did not bleed while probing and had a pocket depth of less than 3 mm.

The demographic data of the participants were recorded in the medical history, including age, sex, and medical records. The participants were assigned to one of four groups according to the severity of their OSA and their periodontal diagnosis, as follows: Group 1 (H), healthy patients: non-periodontitis and non-OSA (*n* = 25); Group 2 (P), periodontitis and non-OSA patients (*n* = 17); Group 3 (OSA), OSA and non-periodontitis patients (*n* = 19); and Group 4 (P-OSA), periodontitis and OSA patients (*n* = 32).

### Oral sample collection

There was no periodontal stimulus performed prior to the collection of the samples (probing, prophylaxis, and calculus removal). Three samples (saliva, subgingival plaque, and gingival sulcus) were taken from the oral cavity of patients. First, they were asked to simply spit into polypropylene tubes containing thioglycolate medium (Oxoid^®^) to collect approximately 1 ml of unstimulated saliva (Nizam et al.,2014). Immediately, the gingival sulcus sample was collected, performing a relative isolation of the area of the tooth of interest with gauze and cotton rolls, and constant drying with cotton swabs to eliminate saliva contamination. In healthy patients, the gingival sulcus sample collection was any area without bleeding, while in patients with periodontitis, this sample was at a site diagnosed with periodontitis. This sample was taken by inserting standardized absorbent papers (Periopapers, Oral Flow^®^, Plainview, NY), approximately 3 mm for 30 s into the periodontal sulcus, which were then put into PBS to elute the Periopaper content by vortexing for 10 s and subsequently transferred to polypropylene tubes containing thioglycolate medium (Gamsiz-Isik et al., 2017). The supragingival plaque was removed with a sterile curette and gauze before obtaining the subgingival plaque sample, which was collected with a curette and introduced into polypropylene tubes containing thioglycolate medium (Nizam et al., 2016). The samples were stored at 4°C during transportation until arrival at the laboratory to be processed.

### Microbial identification by MALDI-TOF-MS

All oral samples were centrifuged at 4,000 ×*g* for 20 min and the pellet was resuspended in 1 ml of thioglycolate medium. A suspension aliquot was cultivated in BBL Columbia Agar™ with 5% sheep blood and another one in Sabouraud agar (Merck^®^), and incubated at 37°C for 2 and 7 days in anaerobiosis and aerobiosis conditions, respectively. At the end of the incubation time, each type of microbial colony was characterized macroscopically and microscopically and identified by mass spectrometry with MALDI-TOF MS, using the MALDI Biotyper^®^ system (Bruker Daltonics Inc., Billerica, MA). For this procedure, the direct method for extracting proteins from microorganisms in plaque was performed applying a small amount of colony on a plaque of the MALDI in duplicate, allowing it to dry at room temperature. Subsequently, 1 μl of formic acid was added to the colonies allowed to dry and then 1 μl of the matrix solution (alpha-cyano-4-hydroxycinnamic acid) was added to extract proteins, mainly ribosomal ones present in high concentrations, and again allowed to dry at room temperature. Once this procedure was performed, the mass spectra were acquired using MALDI-TOF-MS equipment (Microflex from^®^ Burker Daltonik Inc). The mass spectra were analyzed within a range of 2,000 to 20,000 m/z. The MALDI Biotyper version 3.0 library and the MALDI Biotyper version 3.1 software were used for identification considering the cutoff scores ‗1.5 for the genus level and ‗1.7 for the species level. Finally, once the colonies were identified, they were stored at −70°C in thioglycolate medium with 20% glycerol for further studies.

### Statistical analysis

The first part of this research consisted in the characterization and level of the microbiota for each group. Then, the relative abundances of microorganisms were quantified according to Socransky’s microbial complexes (Socransky et al., 1998), adding three categories: yeasts (*Candida* spp.), microorganisms of the oral cavity not classified in the complexes (*Lactobacillus* spp.), and other microorganisms neither classified in the complexes nor associated with specific pathologies in the oral cavity (*Staphylococcus* spp., *Rothia* spp., *Cutibacterium* spp., and *Atopobium* spp.). In the second part, tests were carried out to compare the relative abundance of each category of microorganisms for each type of sample (saliva, subgingival plaque, and gingival sulcus) within each group of patients. The comparison of the relative abundances of microorganisms among the different groups of patients was carried out (*p*-value <0.5). Finally, association tests were performed within each group using the Spearman *r* test (*p*-value <0.5) and a multidimensional grouping analysis according to the abundance of microorganisms common among the groups of patients. The tests GraphPad Prism 9.0.2 (GraphPad Software, California, USA), the free software R 4.01 license GPNU (Free Software Foundation, Boston, USA), and XLSTAT statistical and data analysis solution (Addinsoft, New York, USA) were used.

## Results

The demographic data, medical history and periodontal parameters of the patients are presented in **Table 1**. The ratio of women to men was high in all groups, except in the Group 4 (P-OSA), where there was a higher percentage of men. The periodontal clinical parameters showed statistically significant differences between Group 1 (H) and the other groups of patients (*p* < 0.05) in relation to the percentage of teeth with periodontitis and the percentage of biofilm (**Table 1**).

**Table 1:** Demographic data and clinical characteristics of the groups of patients.

	Group 1 (H) (*n* = 25)	Group 2(P) (*n* = 17)	Group 3 (OSA) (*n* = 19)	Group 4(P-OSA) (*n* = 32)
***Demographic parameters***				
**Age (years), mean (SD)**	46 (14)	41 (10)	50 (13)	49 (11)
**Sex (female) (%)**	80	76	63	34
**Sex (male) (%)**	20	24	37	66
***Systemic conditions***				
**HTN (%)**	12	18	42	19
**HLD (%)**	8	6	11	3
**HT (%)**	8	18	11	19
***Clinical parameters***				
**Teeth present, mean (SD)**	26.0 (3.92)	26.8 (2.22)	23.89 (6.29)	25,22 (5.59)
**Missing teeth, mean (SD)**	5.96 (3.92)	5.23 (2.22)	8.1 (6.29)	6.78 (5.62)
**Percentage of teeth with periodontitis, mean (SD)**	1.94 (3.57)	43.83 (24) *	1.62 (3.15)	38.48 (21.25) *
**Presence of caries (%)**	12	18	5	31
**Percentage of biofilm, mean (SD)**	23 (16)	45.2 (25.95) **	38.6 (20.63) **	40.6 (18.7) **

Group 1 (H) healthy patients, non-periodontitis, and non-OSA; Group 2 (P) periodontitis and non-OSA patients; Group 3 (OSA) OSA and non-periodontitis patients; Group 4 (P-OSA) periodontitis and OSA patients. SD, standard deviation. HTN, arterial hypertension; HLD, hyperlipidemia; HT, hypothyroidism. Significant differences, G1 (S) vs. G2 (P) p ≤ 0.0001*; G1 (S) vs. G4 (PA) p ≤ 0.0001*; G1 (S) vs. G2 (P) **p = 0**.0014**; G1 (S) vs. G3 (P) p = 0.0072**, G1 (S) vs. G4 (P) p = 0.0004**. Multiple **t**-test (multiple comparisons using the Bonferroni–Dunn method).

Regarding the periodontal condition, it was evidenced that 56% of patients in Group 1 (H) had biofilm-induced gingivitis in a reduced periodontium vs. clinical gingival health on an intact periodontium (*p* = 0.000419), followed by stable periodontal disease in a reduced periodontium with a 32% prevalence vs. clinical gingival health on an intact periodontium (*p* = 0.03248). These conditions are considered in healthy patients according to the latest classification of periodontal diseases (Caton et al., 2018). In Group 2 (P), 65% of patients presented stage III periodontitis vs. stage I periodontitis (*p* = 0.0012). In Group 3 (OSA), 79% of patients presented biofilm-induced gingivitis in a reduced periodontium vs. Group 1 (*p* = 0.024). In Group 4 (P-OSA), 81% of the patients presented stage III periodontitis vs. Groups 1 and 3 (*p* < 0.0001). Regarding the diagnosis of OSA according to the degree of severity in Group 3 (OSA), 63% of patients presented mild OSA (mild OSA vs. moderate OSA: *p* = 0.006; mild OSA vs. severe OSA: *p* = 0.023), 16% presented moderate OSA and 21% presented severe OSA, with mild OSA being the most prevalent. However, in Group 4 (P-OSA), 19% of patients had mild OSA, 31% had moderate OSA, and 50% had severe OSA (*p* = 0.02), showing statistically significant differences between Group 3 (OSA) and Group 4 (P-OSA) (*p* = 0.04) (**Table 2**).

**Table 2:** Percentage of patients of each group according to periodontal condition and degree of OSA.

	Group 1 (H) (*n* = 25)	Group 2 (P) (*n* = 17)	Group 3 (OSA) (*n* = 19)	Group 4 (P-OSA) (*n* = 32)	*p*-value*	*p*-value^‡^
	Percentage (%)					
***Periodontal condition***						
Clinical gingival health on an intact periodontium	4	0	5	0	ns	ns
Biofilm-induced gingivitis	8	0	0	0	ns	ns
Biofilm-induced gingivitis in a reduced periodontium	56*	0	79‡	0	0.000419	0.024
Stable periodontal disease in reduced periodontium	32*	0	16	0	0.03248	ns
Periodontitis Stage I	0	6	0	0	ns	ns
Periodontitis Stage II	0	24	0	16	ns	ns
Periodontitis Stage III	0	65*	0	81‡	0.0012	0.0001
Periodontitis Stage IV	0	6	0	3	ns	ns
***Degree of OSA***						
Mild OSA	0	0	63*‡	19	0.006	0.023
Moderate OSA	0	0	16	31	ns	ns
Severe AOS	0	0	21	50*‡	0,02	0,04

Group 1 (H): p-value* of Clinical gingival health on an intact periodontium vs. Biofilm-induced gingivitis in a reduced periodontium and Clinical gingival health on an intact periodontium vs. Stable periodontal disease in reduced periodontium. Group 2 (P): p-value* of Periodontitis Stage I vs. Periodontitis Stage III. Group 3 (OSA): p-value* of Mild OSA vs. Moderate OSA; p-value‡ of Mild OSA vs. Severe AOS, and Clinical gingival health on an intact periodontium vs. Biofilm-induced gingivitis in a reduced periodontium. Group 4 (P-OSA): p-value* of Mild OSA vs. Severe AOS; p-value‡ of Moderate OSA vs. Severe AOS, and Periodontitis Stage I vs. Periodontitis Stage III.

Similarly, when the link between OSA and periodontitis was investigated, it was found that 35% of individuals with periodontitis had mild OSA, 71% had moderate OSA, and 80% had severe OSA. Although the association between OSA and periodontitis did not show statistically significant difference (*p* = 0.2813), the stage III periodontitis was statistically significant with severe OSA (*p* = 0.0157) (**Table 3**).

**Table 3:** Frequency and percentage of the periodontal condition according to apnea diagnosis.

	OSA Diagnosis								
Periodontal diagnosis	Non-OSA		Mild OSA		Moderate OSA		Severe OSA		*p-*value
	Fr	%	Fr	%	Fr	%	Fr	%	
**Non-periodontitis**	25	60	11	65	4	29	4	20	0.2813
**Stage I** **Periodontitis (Mild)**	1	2	0	0	0	0	0	0	0.0157
**Stage II Periodontitis (Moderate)**	3	7	1	6	3	21	1	5	
**Stage III Periodontitis (Severe)**	12	29	5	29	6	43	15	75	
**Stage IV Periodontitis (Advanced)**	1	2	0	0	1	7	0	0	
**Total**	42	100	17	100	14	100	20	100	

Fr: Frequency, %: Percentage; two-way ANOVA, p < 0.05.

### Distribution of complexes and categories of microorganisms

A general descriptive analysis of all groups showed that the relative frequency in the presence of the microorganisms for the purple complex, the categories of *Candida* spp., *Lactobacillus* spp., and others, was higher in saliva and the relative frequency of the other complexes was higher in subgingival plaque (**Table 4**).

**Table 4:** Relative frequencies of the distribution of complexes and categories by samples in the groups of patients.

	Yellow complex	Blue complex	Purple complex	Green complex	Orange complex	Red complex	*Candida* spp.	*Lactobacillus* spp.	Other
**Saliva**	0.32	0.14	**0.54**	0.17	0.28	0.25	**0.50**	**0.71**	**0.54**
**Subgingival plaque**	**0.44**	**0.50**	0.37	**0.50**	**0.45**	**0.75**	0.42	0.16	0.28
**Gingival sulcus**	0.24	0.36	0.09	0.33	0.28	0.00	0.08	0.12	0.18

The bold values means Kruskal-Wallis chi-squared p-value < 0.05.

**Figure 1:**
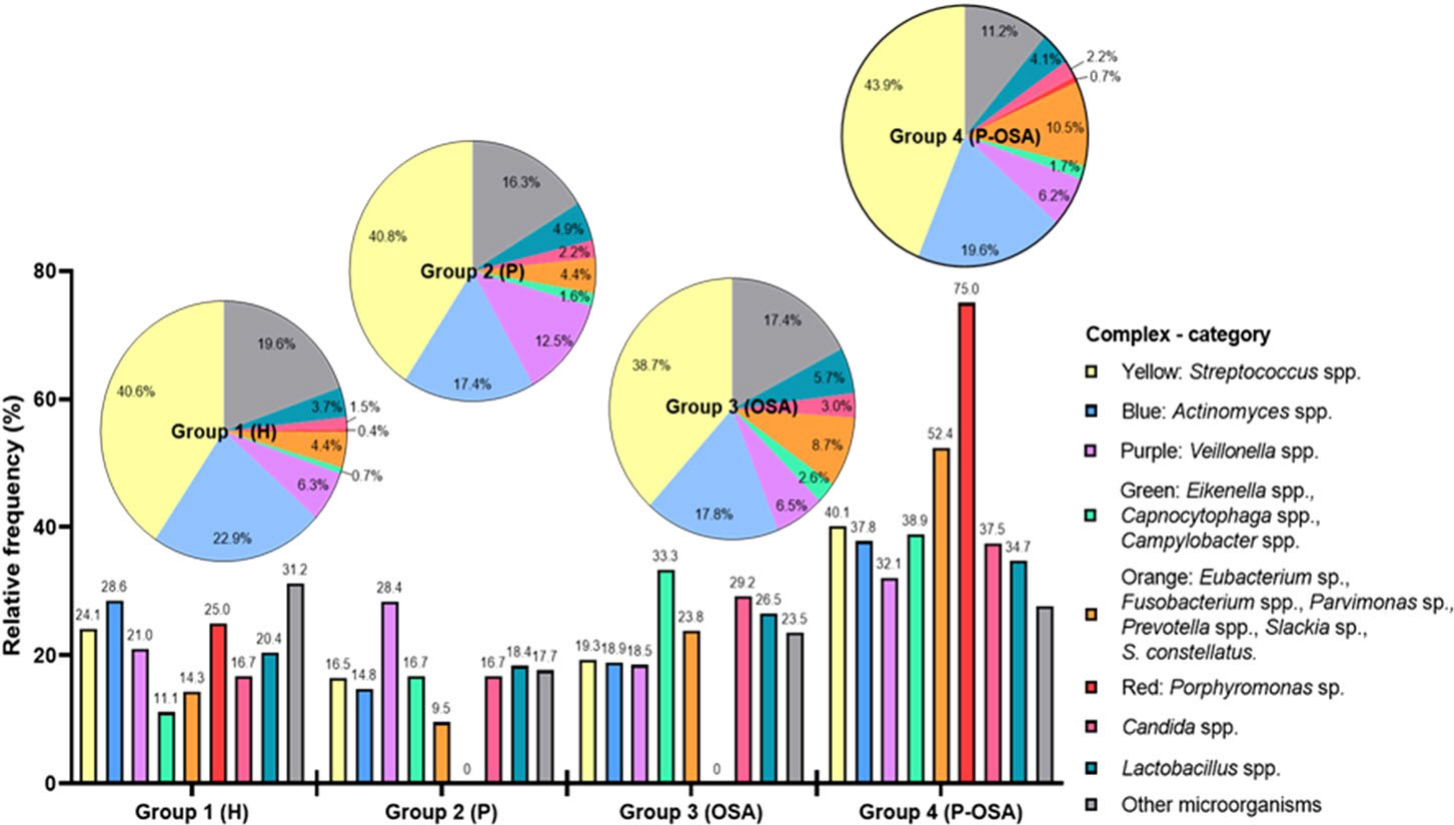
Microbial community composition is grouped by complexes or categories. Bars indicate the percentages of the inter-group relative frequencies of the complexes/categories in group patients. Pie charts show the intra-group distribution percentages of complexes/categories in group patients: Group 1 (H) healthy patients, non-periodontitis and non-OSA; Group 2 (P) periodontitis and non-OSA patients; Group 3: (OSA) OSA and non-periodontitis patients; Group 4 (P-OSA) periodontitis and OSA patients.

The percentages of microorganisms for each complex and category were adjusted to percentages of relative frequency according to the total number of microorganisms identified by complex and category in each group of patients and the total number of microorganisms identified by each group. In this way, the microbial composition inter- and intra-group of patients was analyzed. According to the inter-group analysis (**Figure 1**, bar graph) there is a greater number of microorganisms distributed in all complexes and categories in Group 4 (P-OSA), highlighting the greater presence of microorganisms of the yellow (40.1%), blue (37.8%), purple (32.1%), green (38.9%), orange (52.4%), and red (75%) complexes as well as *Candida* spp. (37.5%) and *Lactobacillus* spp. (34.7%); on the contrary, other microorganisms had a greater frequency in Group 1 (H) (31.2%). Regarding the intra-group analysis, there was a different composition of complexes and categories. The highest frequency of microorganisms of the yellow complex was found in Group 4 (P-OSA) (43.9%); the blue complex had a higher frequency (22.9%) in Group 1 (H); the purple complex had a higher frequency (12.5%) in Group 2 (P); the green complex (2.6%), *Candida* spp. (3.0%), and *Lactobacillus* spp. (5.7%) had a higher frequency in Group 3 (OSA); the orange (10.5%) and the red (0.7%) complexes had a higher frequency of microorganisms in Group 4 (P-OSA); and, finally, other microorganisms (19.6%) were identified more frequently in Group 1 (H).

**Figure 2:**
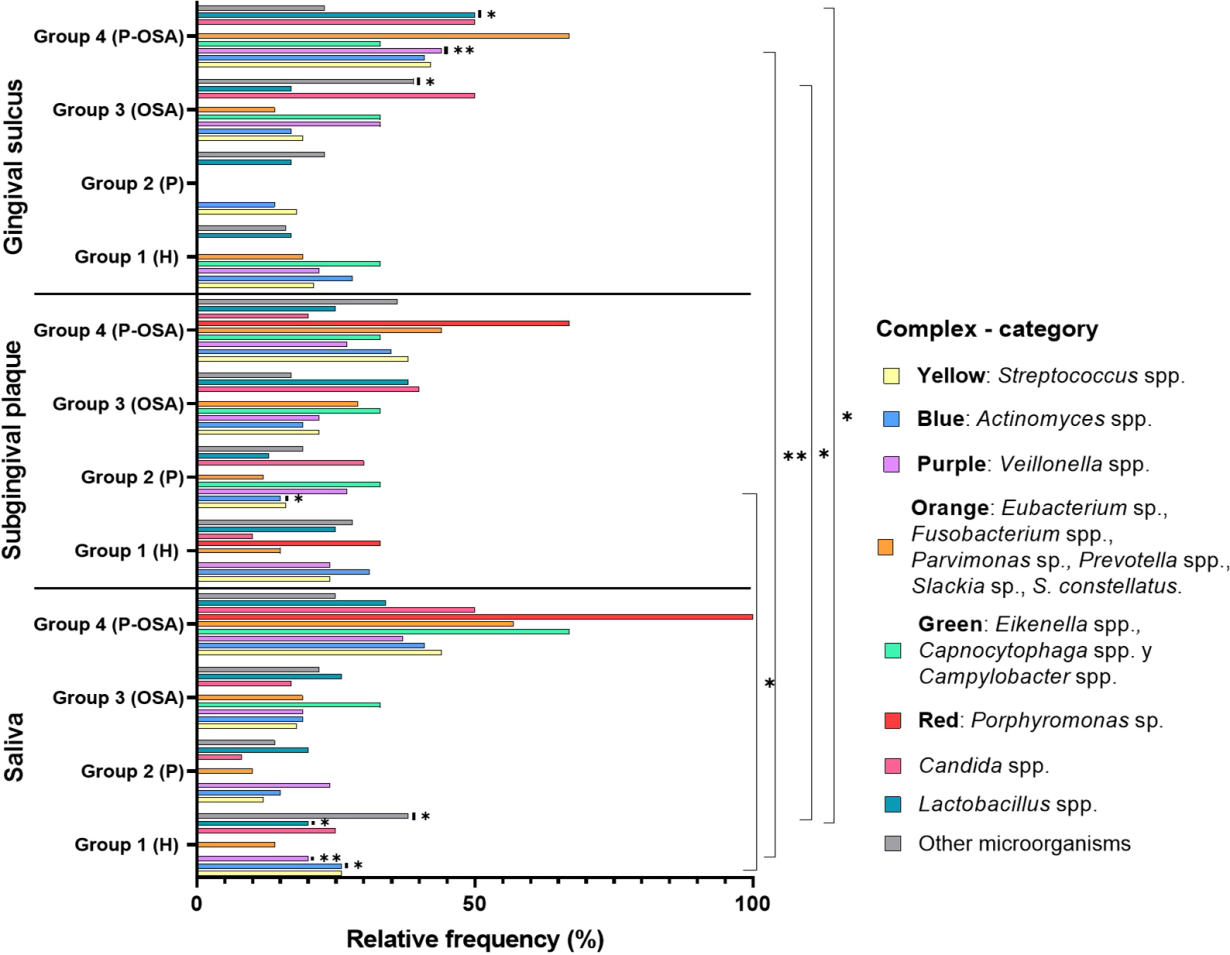
Relative frequencies of the complexes/categories in the oral samples by groups of patients. Significant differences between the relative frequencies of the complexes in each group were evaluated using the Kruskal-Wallis chi-square method; *p-value < 0.05, **p-value < 0.01.

Regarding the distribution of the complexes in each sample (saliva, subgingival plaque, and gingival sulcus), it was evident that the greatest distribution of the complexes and categories was found in saliva and gingival sulcus of Group 4 (P-OSA), except for other microorganisms. On the other hand, there was a greater distribution of all complexes in subgingival plaque, except for *Candida* spp. and *Lactobacillus* spp. in this same group of patients. Additionally, this distribution was not group-dependent either in subgingival plaque or in gingival sulcus. The comparison of the distribution for each complex and the categories in the oral samples in the different groups showed no significant differences for yellow and orange complexes (*p* = 0.1988 and *p* = 0.3045, respectively). However, there were significant differences in the distribution of the blue complex between the Group 1 (H) saliva samples and the Group 2 (P) subgingival plaque sample (*p* = 0.026). In addition, there were significant differences (*p* = 0.009) in the distribution of the purple complex between saliva (Group 1, H) and gingival sulcus (Group 4, P-OSA). Also, there were significant differences (*p* = 0.017) in the distribution of *Lactobacillus* spp. between saliva (Group 1, H) and gingival sulcus (Group 4, P-OSA). The distribution of other microorganisms between the saliva (Group 1, H) and gingival sulcus (Group 3, OSA) was statistically significant (*p* = 0.019) (Kruskal–Wallis chi-square method; *p*-value < 0.05) (**Figure 2**). Additionally, it was determined that *S. salivarius, S. gordonii, S. oralis, A. odontolyticus, A. naeslundii, V. parvula, E. corrodens, P. micra, F. nucleatum, P. melaninogenica, P. gingivalis, C. albicans, L. paracasei*, and *Staphylococcus* spp. were the most frequent species identified in oral samples from all the groups of subjects (**Supplementary Table 1**).

### Association between medical history and cultivable oral microbiota

**Figure 3:**
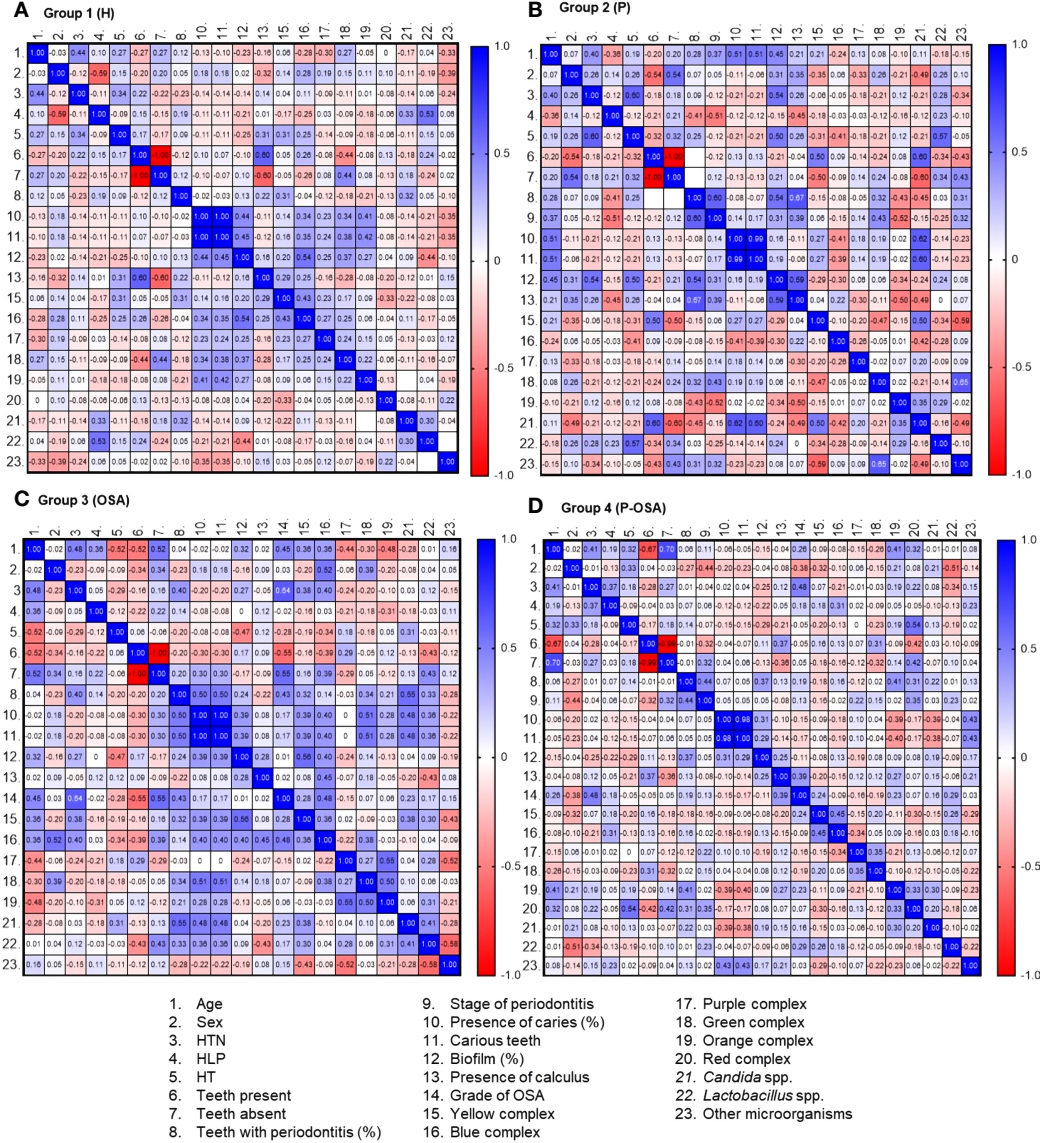
Multicomponent matrix for the correlation of clinical factors and complex/categories of microorganisms present in each of the groups of patients evaluated: **(A)** Group 1 (H), n = 25; **(B)** Group 2 (P), n = 17; **(C)** Group 3 (OSA), n = 19; **(D)** Group 4 (P-OSA), n = 32, using the Spearman's rank correlation coefficient rs > 0.30, p<0.05. The blue tones represent a positive correlation and the red tones represent a negative correlation. HTN, arterial hypertension. HLD, hyperlipidemia. HT: hypothyroidism.

This association was established through the analysis of multicomponent matrices to correlate the comorbidities and oral conditions and the complexes and categories of microorganisms present in the four groups of patients evaluated. The association can be positive (+) or negative (−) according to the Spearman correlation range (*r_s_*). R_s_ values greater than zero in blue tones represent a positive correlation, while *r_s_* values less than zero in reddish tones represent a negative correlation (**Figure 3**). In Group 1 (H), there was a positive, statistically significant correlation between the percentage of biofilm and the presence of caries (*r_s_* = 0.44) and the presence of blue complex (*r_s_* = 0.34), green complex (*r_s_* = 0.34), and orange complex (*r_s_* = 0.41). Also, it is noteworthy that the blue and green complexes are correlated with the percentage of biofilm (*r_s_* = 0.54 and 0.37, respectively), in contrast to *Lactobacillus* spp. and the percentage of the biofilm, which showed a negative correlation (*r_s_* = −0.44). Also, there was a positive correlation between the yellow complex and the blue complex (*r_s_* = 0.43) and a negative correlation between the yellow complex and the red complex, with slight statistical significance (*r_s_* = −0.33) (**Figure 3A**).

In Group 2 (P), there was a positive correlation with statistical significance between age and the presence of caries as with the percentage of biofilm (*r_s_* = 0.51 and 0.45, respectively). In turn, the percentage of biofilm is positively correlated with hypertension, hypothyroidism, and the presence of calculus (*r_s_* = 0.54, 0.50, and 0.59 respectively). Also, the presence of *Candida* spp. was positively correlated with the presence of caries (*r_s_* = 0.62) and the yellow complex (*r_s_* = 0.50). Even though the percentage of teeth with periodontitis, the stage of periodontitis, and the presence of calculus were negatively correlated with the orange complex (*r_s_* = −0.43, −0.52, and −0.50, respectively), they were positively correlated with the green complex and other microorganisms, which likewise presented a positive correlation between them (*r_s_* = 0.65). In addition, there was a positive correlation between the number of teeth absent and the presence of other microorganisms (*r_s_* = 0.43). Other microorganisms have a negative correlation with the presence of *Candida* spp. and the yellow complex (*r_s_* = −0.49 and −0.59, respectively) (**Figure 3B**).

In Group 3 (OSA), the age of the patients had a positive statistically significant correlation with hypertension and the degree of severity of OSA (*r_s_* = 0.48 and 0.45, respectively). In addition, HTN was positively correlated with severity of OSA (*r_s_* = 0.64), the yellow complex (*r_s_* = 0.38), and the blue complex (*r_s_* = 0.40). When analyzing the percentage of teeth with periodontitis, it was possible to identify a positive correlation with the presence of caries (*r_s_* = 0.50), the severity of OSA (*r_s_* = 0.43), and the presence of *Candida* spp. (*r_s_* = 0.55). The presence of caries and number of decayed teeth were positively correlated with the percentage of biofilm (r_s_ = 0.39); the yellow (r_s_ = 0.39), blue (*r_s_* = 0.40), and green *r_s_* = 0.51) complexes; and *Candida* spp. (*r_s_* = 0.48). In turn, the percentage of biofilm correlated positively with the presence of the yellow and blue complexes (*r_s_* = 0.56 and 0.40, respectively). The blue complex was correlated positively with the presence of calculus and the severity of OSA (*r_s_* = 0.45 and 0.48, respectively). When analyzing the relationship between the presence of the complexes, it was evidenced that the yellow complex is positively correlated with *Candida* spp. with a slight statistical significance (*r_s_* = 0.38). A similar correlation was given between the blue and the green complexes (*r_s_* = 0.38), which had a positive correlation with the orange complex (*r_s_* = 0.50). The latter complex was positively correlated with the purple complex (*r_s_* = 0.55), and *Candida* spp. and *Lactobacillus* spp. presented a positive correlation (*r_s_* = 0.41). Other microorganisms presented a negative correlation with the presence of the yellow complex (*r_s_* = −0.43), purple complex (*r_s_* = −0.52), and *Lactobacillus* spp. (*r_s_* = −0.58) (**Figure 3C**).

In Group 4 (P-OSA), the age of the patients was correlated positively with the HTN (*r_s_* = 0.41), absence of teeth (v = 0.70), the orange complex (*r_s_* = 0.41), and the red complex (*r_s_* = 0.32). Similarly, it was found that HTN has a positive correlation with HLD (*r_s_* = 0.37) and the severity of OSA (*r_s_* = 0.48). The lack of teeth in patients with periodontitis and OSA has been shown to be strongly connected with periodontitis stage (*r_s_* = 0.32) and the presence of red complex bacteria (r_s_ = 0.42). On the other hand, the percentage of teeth with periodontitis was correlated with the percentage of biofilm (*r_s_* = 0.37), the presence of the orange complex (*r_s_* = 0.41), and slightly with the red complex (*r_s_* = 0.31). In this group of patients, the presence of calculus was positively correlated with the severity of OSA (*r_s_* = 0.39) and the stage of periodontitis was positively correlated with the presence of the red complex (*r_s_* = 0.35). About the correlation of the presence of the complexes, there was a positive correlation between the yellow and blue complexes (*r_s_* = 0.45), the purple and green complexes (*r_s_* = 0.35), the orange and red complexes (r_s_ = 0.33), and the orange complex with *Candida* spp. (*r_s_* = 0.30) (**Figure 3D**).

### Prevalence of complexes and categories

**Figure 4:**
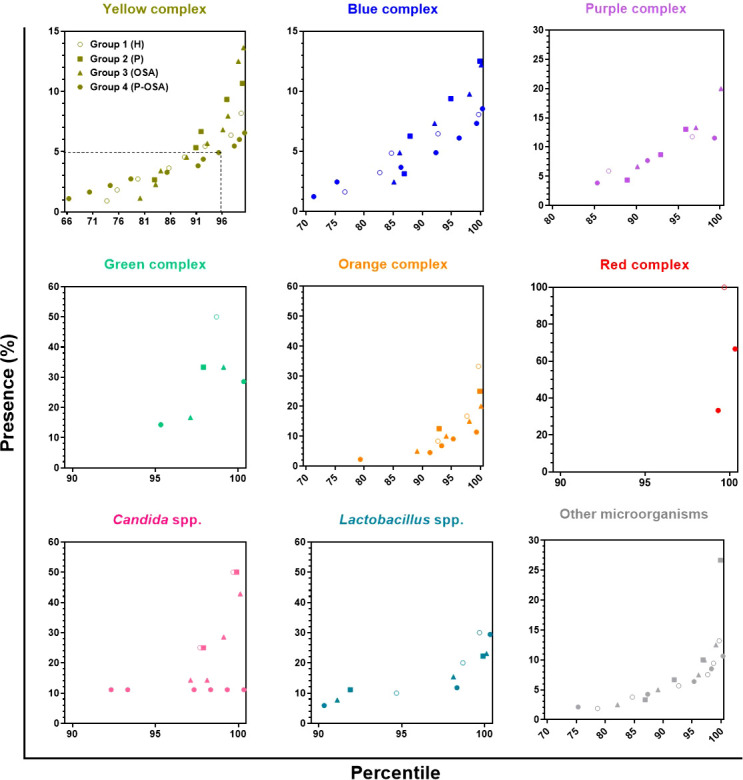
Percentile graph of the percentage of presence of complexes/categories in each of the groups of participants evaluated. Each point represents the presence (%) of the complex/category in a patient. The x-axis represents the percentiles, the y-axis the presence (%) of the complexes/categories, and the dotted line in the first panel indicates that 95% of the participants in Group 4 (P-OSA) have less than 5% presence of the yellow complex, on the other hand, 5% of the subjects of this same group exhibit > 5% presence of the yellow complex.

**Figure 4** presents the percentile plots that indicate the prevalence of the complexes and categories in each group of patients evaluated. This figure indicates that the yellow complex was detected in 27% of the patients of Group 1 (H), who harbored up to 8% of the microorganisms of this complex. In Group 2 (P), it was detected in 18% of the patients, who harbored up to 11% of the microorganisms of this complex. In Group 3 (OSA), it was detected in 21% of the patients, who presented up to 14% of the microorganisms of the complex. In Group 4 (P-OSA), it was detected in 35% of the patients, who presented up to 7% of the microorganisms of the yellow complex. The blue complex was detected in 24% of the patients of Group 1 (H), 14% of the patients of Group 2 (P), 16% of the patients of Group 3 (OSA), and 30% of the patients of Group 4 (P-OSA), who presented up to 8%, 13%, 12%, and 9% of the microorganisms of this complex, respectively. The purple complex was detected in 14%, 12%, 11%, and 16% of the patients of Group 1 (H), Group 2 (P), Group 3 (OSA), and Group 4 (P-OSA), respectively, who presented respectively up to 12%, 13%, 20%, and 11% of the microorganisms of this complex. The green complex was detected in 2%, 3%, 4%, and 6% of the patients of Group 1 (H), Group 2 (P), Group 3 (OSA), and Group 4 (P-OSA), respectively, who presented up to 50%, 33%, 33%, and 29% of the microorganisms of this complex, respectively. The orange complex was detected in 8% of the patients of Group 1 (H) and Group 2 (P), and in 12% and 28% of the patients of Group 3 (OSA) and Group 4 (P-OSA) respectively, who presented up to 33%, 25%, 20%, and 11% of the microorganisms of this complex, respectively. The red complex was detected in 1% of Group 1 (H) and 2% of Group 4 (P-OSA) patients. The category *Candida* spp. was detected in 3% of the patients of Group 1 (H) and Group 2 (P), which housed up to 50% of the yeasts of this category, and on the other hand, this category was detected in 4% of the patients of Group 3 (OSA) and 9% of the patients of Group 4 (P-OSA), which presented up to 42% and 11% of the yeasts of this category, respectively. The category *Lactobacillus* spp. was detected in 6%, 9%, 10%, and 11% of the patients of Group 1 (H), Group 2 (P), Group 3 (OSA), and Group 4 (P-OSA), respectively, who housed up to 30%, 22%, 23%, and 29% of *Lactobacillus* spp., correspondingly. Finally, the category other microorganisms was detected in 22%, 14%, 19%, and 26% of the patients of Group 1 (H), Group 2 (P), Group 3 (OSA), and Group 4 (P-OSA), who presented up to 13%, 27%, 12.5%, and 10.6% of the microorganisms in this category, respectively (**Figure 4**). All complexes and categories of *Candida* spp., *Lactobacillus* spp., and other microorganisms were more prevalent in Group 4 (P-OSA); however, no statistically significant differences were found.

### Multidimensional scaling analysis

**Figure 5:**
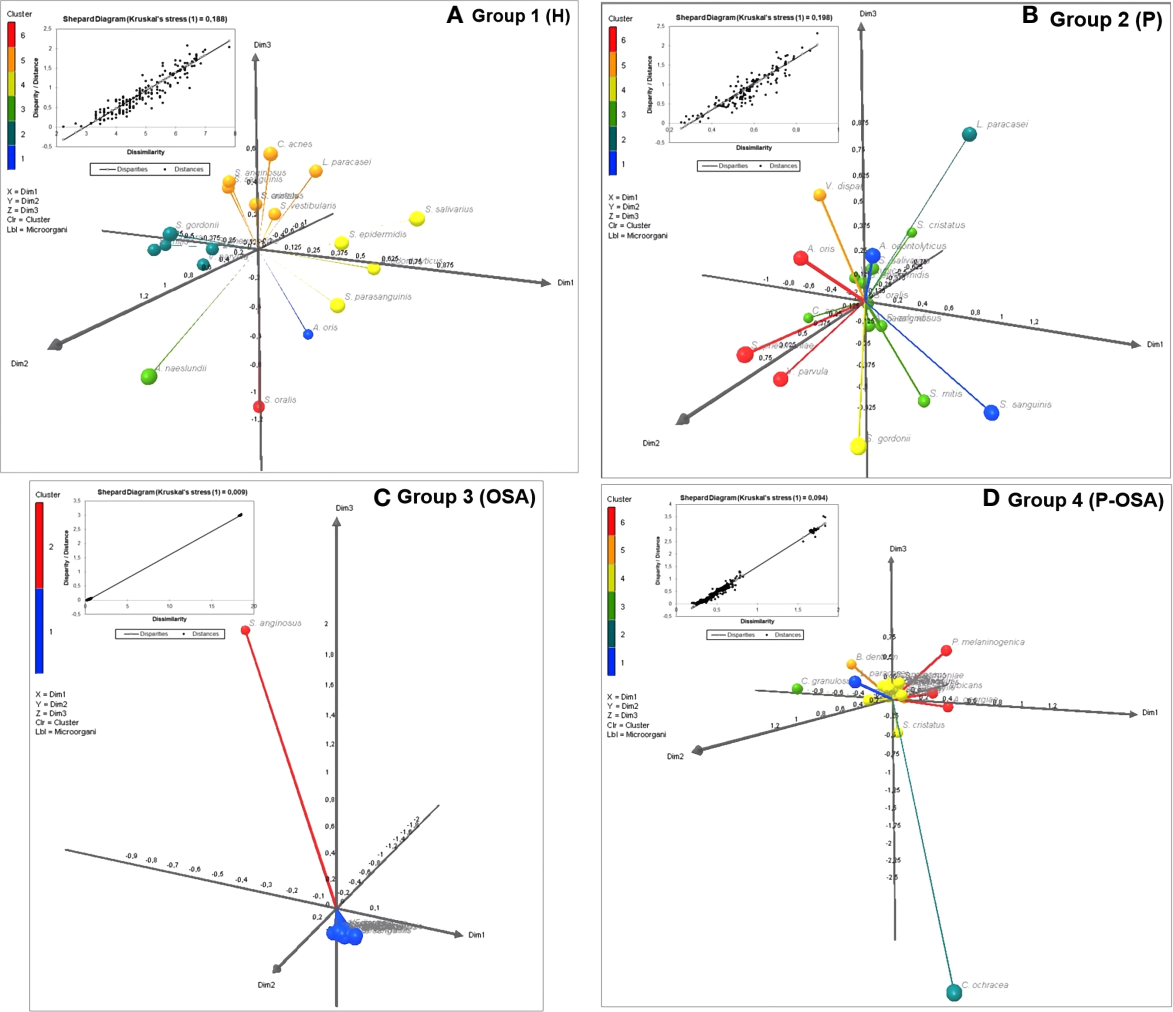
Multidimensional Scaling (MDS) based on Proximity Matrices (Euclidean Distance) and Kruskal Stress for the three-dimensional visualization (XLSTAT3DPlot) of the consortia made up of the most common microorganisms by group of patients: **(A)** Group 1 **(H)**; **(B)** Group 2 (P); **(C)** Group 3 (OSA); **(D)** Group 4 (P-OSA).

To show the differences between the consortia of the most common microorganisms present in each group of patients, we performed a multidimensional scaling (MDS) analysis. This analysis was established according to the dissimilarity (distance matrix) of the microorganisms present in each consortium, through the stress adjustment measure that is based on the differences between the predicted and actual distances and visualized in 3D plots (**Figure 5**). Six clusters formed the consortiums of microorganisms in both the group of healthy patients (Group 1) and the group of patients with periodontitis (Group 2), which showed the greatest intra-group dissimilarity (Kruskal stress = 0.188 and 0.198, respectively); on the other hand, two clusters formed the consortium of microorganisms in patients with OSA (Group 3) and six clusters formed the consortium of microorganisms in patients with periodontitis and with OSA (Group 4), which showed the smallest intra-group dissimilarity (Kruskal stress = 0.009 and 0.094, respectively).

In Group 3, dimension 3 was defined by *Streptococcus anginosus* (yellow complex) and dimensions 1 and 2 were composed of the rest of the common microorganisms; this means that the presence of *S. anginosus* could be a distinctive marker within the patients of this group, in terms of the most common microorganism found. On the other hand, in Group 4, another microorganism was found as the most common or distinctive marker: *Capnocytophaga ochracea* (green complex), which defined dimension 3 but with a negative modulation, while the positive axis of dimensions 1 and 2 was differentiated by *Actinomyces georgiae* (blue complex), *Prevotella melaninogenica* (orange complex), and *C. albicans*, the three of them forming a cluster, and *Lactobacillus paracasei* predominated in the positive axis of dimension 3.

## Discussion

This is the first report regarding the characterization of the oral microbiota in patients with periodontitis associated with OSA using MALDI-TOF MS as a tool for rapid identification of oral microorganisms. Additionally, the differences in the oral microbiota between individuals with and without periodontitis, and with and without OSA had not been reported before. The present study showed that there are more cases of stage III periodontitis in patients with severe OSA. Our results are similar to the findings reported by Gamsiz-Isik et al. (2017) and support the hypothesis that there is a higher prevalence of periodontitis in patients with OSA (Gunaratnam et al., 2009; Ahmad et al., 2013; Seo et al., 2013; Cuervo et al., 2016; Gamsiz-Isik et al., 2017; Latorre et al., 2018).

When determining the periodontal parameters of the patients evaluated in this study, a higher percentage of biofilm in patients with periodontitis, with OSA, and with both conditions was detected in opposition to healthy patients (*p* < 0.01). This finding is consistent with the significant relationship between the presence of OSA and the percentage of biofilm reported by Loke et al. (2015) and by Gamsiz-Isik et al. (2017). The evidence suggests that the colonization of a dysbiotic microbiota favored by oral dryness increases CO_2_ levels and decreased O_2_, and those three are the main factors involved in the pathophysiology of OSA (Nizam et al., 2016), which, in turn, affects bone remodeling in patients with periodontitis.

In the present study, a characterization of the microbiota established in patients with OSA and periodontitis was carried out. It was possible to determine the highest relative frequencies of all of the Socransky’s complexes in the subgingival plaque, except the purple one, while the purple complex, *Candida* spp., *Lactobacillus* spp., and other microorganisms presented the highest relative frequencies in saliva. Although it was evidenced that the complexes and categories of microorganisms are present in all the groups evaluated, a greater microbial diversity was demonstrated in the patients with both conditions (OSA and periodontitis). All groups showed different percentages of microorganisms according to the sample evaluated (saliva, subgingival plaque, or gingival sulcus), raising a possible “modulation” of the microorganisms depending on the state of health or disease (Socransky and Haffajee, 2005; Curtis et al., 2020).

The use of the MALDI-TOF MS, which only identifies cultivable microorganisms, was one of the study’s limitations. This method might have an impact on the findings and might have omitted the presence of other microorganism species. Additionally, these findings should be validated in further studies with a bigger sample and in other populations.

Despite the limitations in having carried out microbial identification by MALDI-TOF MS, it is important to highlight that our results are comparable to those of prior studies and support the hypothesis that OSA influences the colonization of a potentially infectious dysbiotic microbiota in the oral cavity, as other authors have proposed. Nizam et al. (2016) determined a marked increase in Gram-negative bacteria in dental plaque samples in patients with severe OSA and periodontal disease and also reported Gram-positive bacteria in dental plaque in severe cases of OSA (Nizam et al., 2016), whereas Chen et al. (2021) determined that well-known periodontal pathogen species do not significantly increase their relative abundance in patients with OSA; however, the salivary microbial community structure was altered, affecting the interactions of different bacteria in the pathogenesis process of periodontitis in patients with OSA, along with an increase of *Prevotella* spp. In the present study, we identified the most common microorganisms in patients with both periodontitis and OSA (P-OSA), highlighting the presence of *A. georgiae, P. melaninogenica, L. paracasei , and C. albicans*, as potential distinctive markers for both diseases. These results might explain the presence of periodontitis in patients with OSA.

Concerning the association of clinical factors and the oral microbiota characterized in each group of patients, the relationship between the percentage of biofilm and other oral pathologies such as caries was evidenced, given the presence of microorganisms mainly of the yellow and blue complexes in healthy patients. This finding is consistent with previous reports in the literature (Colombo and Tanner, 2019; Valm, 2019). Despite not fulfilling all the parameters to be classified into the group of periodontitis according to the new classification adopted since 2018 (Caton et al., 2018), the presence of teeth with periodontitis was detected in these patients, and it was related to the bacteria of the yellow complex and *Candida* spp. These data are relevant considering that different authors have reported the colonization of yeasts of the genus *Candida*, especially *C. albicans*, in periodontal pockets of patients with severe chronic periodontitis (Canabarro et al., 2013), and the prevalence of these species was 50% and 60% in patients with aggressive and chronic periodontitis, respectively (Vieira Colombo et al., 2016). These previous results suggest that *C. albicans* plays a role in the progression of conditions such as periodontitis, given its known pathogenic power to invade oral epithelial tissue, form hyphae, secrete proteinases, and interact with commensal streptococci to synergistically promote their virulence (Martin et al., 2011; Xu et al., 2014; Belibasakis et al., 2015; Charalampakis and Belibasakis, 2015; Chevalier et al., 2017; Mombelli, 2018).

In recent decades, there has been an increase in reports demonstrating the bidirectional risk between periodontitis and systemic diseases such as hypertension (Latorre et al., 2018), CVDs (Bui et al., 2019), hypothyroidism, metabolic syndromes (Jaramillo et al., 2017), and diabetes (Zhou et al., 2013; Escobar et al., 2014; Genco and Borgnakke, 2020). In older patients, this risk was confirmed by the correlation of the percentage of biofilm detected in patients of Group 2 (P) with age of patients (*r_s_* = 0.45, *p* = 0.035), and with diseases such as hypertension (*r_s_* = 0.54, *p* = 0.0162) and hypothyroidism (*r_s_* = 0.50, *p* = 0.0023). Additionally, the cases of caries present in this group of subjects were associated with the presence of *Candida* spp. and, in turn, with the bacteria of the yellow complex, contributing to the hypothesis that yeasts of the genus *Candida* can behave as a primary colonizer in dysbiotic biofilms (Janus et al., 2017). The percentage of teeth with periodontitis was associated with the percentage of biofilm and the presence of calculus. These results confirm that the formation of polymicrobial consortia is one of the main factors contributing to the risk of periodontitis. In Group 2 (P), periodontitis was mainly associated with the presence of green complex bacteria, recognized in mature pathogenic biofilms where environments with low oxygen concentrations are produced (Haffajee et al., 2008). Therefore, these findings support the role of these bacteria as indicators of ecological changes in the subgingival biofilm during the progression of periodontitis (Henne et al., 2014). Likewise, the category of “other microorganisms”, in which those that are not usually identified as oral pathogens were grouped, was also associated with periodontitis. These results confirm the prevalence of other potentially pathogenic species of clinical relevance in the oral cavity as was evidenced by Vieira Colombo et al. (2016) who showed the microbiota of the subgingival plaque of individuals with different states of periodontitis, highlighting the presence of bacteria of the genera *Neisseria*, *Peptostreptococus*, *Pseudomonas*, *Clostridium*, and *Enterobacteria*, among others (Vieira Colombo et al., 2016).

OSA is a risk factor for CVDs, as it increases morbidity and mortality in patients with arterial hypertension (HTN), coronary heart disease, atrial fibrillation, and heart failure (Zhou et al., 2013; Genco and Borgnakke, 2020). The results of our study showed that the severity of OSA in Group 3 was associated with hypertension and with the percentage of teeth with periodontitis. Thus, it is necessary to emphasize that the presence of teeth with periodontitis in this group is correlated with the severity of OSA, caries, and the presence of *Candida* spp., establishing a possible association between the infectious processes in the periodontium and the presence of microorganisms such as *Candida* spp. that are different from the usual periodontopathogens.

The cause–effect relationship between periodontitis and OSA, however, is not fully understood. Several mechanisms have been proposed, involving genetic, immunological, and microbiological factors (Nizam et al., 2014; Al-Jewair et al., 2015). Our findings suggest that there is an association between risk factors such as HTN, HLD, and HT and patient age, which supports the previously reported bidirectional relationship of both diseases (Gunaratnam et al., 2009; Keller et al., 2013; Seo et al., 2013; Sanders et al., 2015; Cuervo et al., 2016; Latorre et al., 2018). Additionally, it was evident that the bacteria of the orange complex participate in this association, together with the red complex, which, in turn, was associated with the loss of teeth and the stage of periodontitis. In the same way, both complexes were associated with the formation of the dysbiotic biofilm (Lamont and Hajishengallis, 2015). While it is true that the presence of bacteria considered periodontopathogenic was associated with the presence of periodontitis in patients with OSA, it is important to note that the presence of *Candida* spp. was more prevalent in patients with periodontitis, and in addition, these yeasts were mainly associated with the bacteria of the orange complex. Then, we propose a new hypothesis: in the presence of both diseases, these opportunistic microorganisms may have a behavior that allows the colonization of periodontopathogenic bacteria. It has been demonstrated through *in vitro* studies that yeasts such as *C. albicans* modulate the microenvironment, causing anoxic conditions, favoring the growth of strictly anaerobic bacteria, and synergistically promoting the formation of dysbiotic biofilms (Diaz et al., 2012; Fox et al., 2014; Sztukowska et al., 2018).

Even if OSA can favor the predisposing factors of periodontitis by the presence of *P. melaninogenica* and *C. albicans* increasing the severity of the periodontal disease, questions arise as to whether the inhibition of these microorganisms can control the pathogenicity of biofilm associated with the conditions evaluated. These findings suggest that antimicrobial strategies for treating periodontitis in patients with OSA can be tested *in vitro* using polymicrobial biofilms.

## Conclusions

*Men had a higher prevalence of stage III periodontitis and severe OSA.
*There was a greater diversity of microorganisms in the oral samples evaluated from the patients with periodontitis and OSA, and the differences in the percentages of the presence of each complex and category were notorious.
*The association between periodontitis and OSA was evidenced by sharing risk factors such as comorbidities and bacteria of the orange and red complexes associated with yeasts such as *Candida* spp.
*Periodontal disease in patients showed positive and similar correlations among yellow, blue, orange, green, and purple bacteria complexes, *P. melaninogenica*, *C. albicans*, and the different oral microenvironments, indicating that the presence of *P. melaninogenica* and *C. albicans* should be considered in the prevention and treatment of this disease.

## Data availability statement

The raw data supporting the conclusions of this article will be made available by the authors, without undue reservation.

## Ethics statement

This study was reviewed and approved by Comite de etica, Facultad de Odontologia, Pontificia Universidad Javeriana. The patients/participants provided their written informed consent to participate in this study.

## Author contributions

MT-C, LO, and CP-G conceived and designed the study. MT-C and EH-D performed the statistical analysis. MT-C and HC-J performed the microbial identification. NA-J and DM-V conducted the periodontal examinations. CL-U and FE-A performed and supervised periodontal examinations. JV-P contributed to data analysis. PH-M practiced medical checks and the polysomnographic study. MT-C wrote the manuscript and prepared the tables and figures. MC, NR-M, LO, and CP-G reviewed and edited the manuscript. All authors contributed to the article and approved the submitted version.

## Funding

Pontificia Universidad Javeriana-sede Bogotá financial support to the project “Characterization of the microbiota associated with obstructive sleep apnea in patients with periodontitis” (project number 008398).

## Acknowledgments

We thank the *Pontificia Universidad Javeriana-sede Bogotá* for the financial support to the project “Characterization of the microbiota associated with obstructive sleep apnea in patients with periodontitis” (project number 008144), and we greatly appreciate the support given by the Training Agreement for postgraduate studies of professors of the *Pontificia Universidad Javeriana* for their doctoral training. We also thank all the participants who were part of this project as patient donors of the oral samples.

## Conflict of interest

The authors declare that the research was conducted in the absence of any commercial or financial relationships that could be construed as a potential conflict of interest.

## Publisher’s note

All claims expressed in this article are solely those of the authors and do not necessarily represent those of their affiliated organizations, or those of the publisher, the editors and the reviewers. Any product that may be evaluated in this article, or claim that may be made by its manufacturer, is not guaranteed or endorsed by the publisher.
