# Supplementary material for: Patients with obstructive sleep apnea can favor the predisposing factors of periodontitis by the presence of P. melaninogenica and C. albicans, increasing the severity of the periodontal disease
Source: Front Cell Infect Microbiol. 2022 Sep 15;12:934298. doi: 10.3389/fcimb.2022.934298 (PMC9519896; doi:10.3389/fcimb.2022.934298)
Supplement: Supplementary file 1 [file Table_1.docx]

| TABLE S1 \| Most frequent and common species of microorganisms in oral cavity samples. | | | | | | |
| --- | --- | --- | --- | --- | --- | --- |
| **Complex - category** | **Group** | **Saliva** | | **Subgingival plaque** | | **Gingival sulcus** |
| **1. Yellow** | **G1 (H)** | | *S. salivarius* | | *S. parasanguinis* | *S. pneumonie* |
|  | **G2 (P)** | |  |  | *S. anginosus* | *S. oralis* |
|  | **G3 (OSA)** | |  |  | *S. gordonii* |  |
|  | **G4 (P-OSA)** | |  |  |  |  |
| **2. Blue** | **G1 (H)** | | 1. *odontolyticus* | | *A.oris* | *A. naeslundii* |
|  | **G2 (P)** | |  |  | *A.naeslundii* |  |
|  | **G3 (OSA)** | |  |  | *A.odontolyticus* | *A. odontolyticus* |
|  | **G4 (P-OSA)** | |  |  | *A.oris* | *A. oris* |
| **3. Purple** | **G1 (H)** | | *V. parvula* | | *V. parvula* | *V. dispar* |
|  | **G2 (P)** | | *V. atypica* | |  | *-* |
|  | **G3 (OSA)** | | *V. parvula* | |  | *V. parvula* |
|  | **G4 (P-OSA)** | |  |  |  |  |
| **4. Green** | **G1 (H)** | | *-* | | *-* | *C. concisus* |
|  | **G2 (P)** | | *-* | | *E. corrodens* | *-* |
|  | **G3 (OSA)** | | *E. corrodens* | | *C. ochracea* | *C. gingivalis* |
|  | **G4 (P-OSA)** | |  |  |  |  |
| **5. Orange** | **G1 (H)** | | *P. micra* | | *P. micra* | *P. micra* |
|  | **G2 (P)** | | *F. nucleatum* | | *P. micra* | *S. constellatus* |
|  | **G3 (OSA)** | |  |  | *F. nucleatum* |  |
|  | **G4 (P-OSA)** | | *P. micra* | | *P. melaninogenica* | *P. melaninogenica* |
| **6. Red** | **G1 (H)** | | *-* | | *P. gingivalis* | *-* |
|  | **G2 (P)** | | *-* | | *-* | *-* |
|  | **G3 (OSA)** | | *-* | | *-* | *-* |
|  | **G4 (P-OSA)** | | *P. gingivalis* | | *P. gingivalis* | *-* |
| **7. *Candida* spp.** | **G1 (H)** | | *C. albicans* | | *C. albicans* | *-* |
|  | **G2 (P)** | |  |  |  | *-* |
|  | **G3 (OSA)** | |  |  |  | *C. albicans* |
|  | **G4 (P-OSA)** | |  |  |  | *C. glabrata* |
| **8. *Lactobacillus* spp.** | **G1 (H)** | | *L. paracasei* | | *L. paracasei* | *L. gasseri* |
|  | **G2 (P)** | | *L. fermentum* | |  | *L. paracasei* |
|  | **G3 (OSA)** | | *L. paracasei* | |  |  |
|  | **G4 (P-OSA)** | |  |  |  | *L. salivarius* |
| 9. Other microorganisms | **G1 (H)** | | *Staphylococcus* spp. | | *Rothia* sp. | *Staphylococcus* spp*.* |
|  | **G2 (P)** | |  |  | *Atopobium* sp*.* |  |
|  | **G3 (OSA)** | |  |  | *Cutibacterium* sp. | *Rothia* sp*.* |
|  | **G4 (P-OSA)** | |  |  | *Alloscardovia* spp*.* | *Staphylococcus* spp*.* |
